# Rebirth of the crippling illness: polio

**DOI:** 10.1097/MS9.0000000000000286

**Published:** 2023-03-27

**Authors:** Rana U. Ahmad, Aisha Shabbir, Laibah A. Khan, Muhammad F. Ashraf

**Affiliations:** Internal Medicine, Mayo Hospital, Anarkali, Lahore, Punjab, Pakistan

*Dear Editor*,

Polio is a deadly infection, mostly affecting children below 5 years of age^1^. It is transmitted via fecal–oral transmission and begins as a series of nonspecific flu-like symptoms, usually with no progression of the disease. However, in a small subset of the population, the virus can lead to acute flaccid paralysis of the lower limbs. Respiratory failure and death occur in 5–10% of paralyzed individuals^1^. Polio spread like wildfire in the 20th century, with 27 000 people paralyzed and 6000 dead in the summer of 1916 in the United States alone^2^. Continued vaccination efforts led to a 99% decline in polio cases from 1988 to 2021, limiting the disease to only two countries^1^. Unfortunately, the polio virus is back again in multiple developed and underdeveloped countries, where it has been completely eradicated for the past two decades.

The two vaccines used for polio are the killed vaccine (Salk) and the oral live attenuated (Sabin) vaccine. There have been rare cases of mutations occurring in the Sabin vaccine leading to virulence and, ultimately, vaccine-derived poliovirus (VDPV), especially in areas of low vaccine coverage^3^. Although the Sabin vaccine has been discontinued from use in the UK since 2004, VDPV was detected in sewage samples in North and East London on 22 June 2022^4^, followed by the detection of the virus in sewage from Jerusalem, Israel^3^. Recently, a case of acute flaccid paralysis has emerged in New York in an unvaccinated individual^3^. This is the first poliovirus case in the US since 2013^5^. It is reported that all of these three patients in different countries belong to the same community which is reluctant to the polio vaccination. The anti-vaccine movement is a growing concern in developed countries, especially among conservative communities^6^.

With the current political instability and war in Ukraine, there has been a rise in the number of polio cases. Numerous reasons led to the emergence of both wild-type and VDPV cases, including the economic crisis, military conflict, the decline in three-dose polio coverage, low perceived risk, and a media-induced rise in the anti-vaccine movement^7^. The risk of polio will only increase, rendering younger children particularly susceptible. In 2021, a case of acute flaccid paralysis caused by the polio virus was reported in Malwai after almost 30 years. This strain was similar to the one reported in the province of Sindh, Pakistan, in October 2019, showing the imported status of this case. Mozambique has also announced similar developments after almost 30 years^8^. These developments are probably related to a decrease in the quality of polio surveillance due to the ongoing coronavirus disease 2019 (COVID-19) pandemic.

There are only two countries in the world from where the wild-type polio virus has never been eradicated – Pakistan and Afghanistan. Pakistan had a surge of polio-infected individuals in 2019, during the COVID-19 pandemic, and though the numbers decreased to an all-time low in 2021, they are again on the rise as of 2022. There are many reasons why the infection persists, like vaccine hesitancy among different cultural populations, poor security of teams working for polio vaccination, prevailing misinformation, and underdeveloped infrastructure to reach far-flung areas, among others. Just recently, gunmen in northwest Pakistan murdered a healthcare worker and two security personnel. There is distrust in the rural population, with myths about microchips and infertility prevalent^9^.

With the prolonged political instability in Afghanistan, there are multiple barriers to immunization, including illiteracy, misinformation, multiple inaccessible areas, huge population movement, and parental mistrust. There is a widespread belief that it is not allowed in Islam and that it is a Western conspiracy used to sterilize children. Furthermore, following the COVID-19 pandemic, most healthcare vaccination facilities were closed, and the vaccine stores ran extremely low due to travel bans, leading to polio cases in previously polio-free provinces^10^. The Taliban’s takeover of Afghanistan in 2021 threatened to put a halt to polio eradication progress, keeping in view their earlier policies of resistance since 2018. However, vaccination has resumed in the country, even in areas that had been closed off by the Taliban, such as southern Afghanistan^11^.

As all developed countries have been using inactivated polio vaccines exclusively, almost for the past two decades meaning that the recently reported cases in developed countries are derived from mutated strains of oral polio vaccine imported via travelers. To effectively eradicate the polio virus, we need to make new and effective oral polio vaccines that would not lead to lethal mutations, preventing the re-activation process. In the meantime, the use of inactivated polio vaccines should be made sure throughout the world, especially in the endemic areas, as they are the ultimate source of spread to all other countries. Furthermore, the spread of the virus can be limited by using modern sewage systems, wastewater management, and providing easy access to purified water for everyone.

Continuing the ongoing vaccination and polio-free programs in every area of the world and also preventing the importation of the virus to virus-free areas are the best ways to narrow down and fully eradicate this disease. Coordinated strategies involving the governments of Pakistan and Afghanistan, especially the tribal leaders of highly endemic areas, can help in carrying out effective vaccination campaigns and eliminating all the cases from newly affected areas. An effective worldwide media campaign is necessary to rectify the misunderstandings about vaccination, control the spread of misinformation, cancel out conspiracies, and propagate all the necessary information about this preventable disease.

## Ethical approval

Not required.

## Consent

Not required.

## Source of funding

None.

## Author contribution

All authors contributed toward data analysis, drafting, and revising the paper, gave final approval of the version to be published, and agreed to be accountable for all aspects of the work.

## Conflicts of interest disclosure

There are no conflicts of interest.

## Research registration unique identifying number (UIN)

1. Name of the registry: none.
2. Unique identifying number or registration ID: none.
3. Hyperlink to your specific registration (must be publicly accessible and will be checked): none.

## Guarantor

Rana Uzair Ahmad.

## Provenance and peer review

Not commissioned, externally peer-reviewed.
